# Seven‐Year Delayed Diagnosis of a Giant Suprasellar Arachnoid Cyst Presenting With Progressive Gait Disturbance: Successful Endoscopic Ventriculocystocisternostomy—A Case Report

**DOI:** 10.1002/ccr3.73681

**Published:** 2026-10-04

**Authors:** Zolfiqar Ibrahem, Abdulrahman Almjersah, Abdullah Mouselli, Iskandar Ismail Saleh, Basel Alromhain

**Affiliations:** ^1^ Department of Neurosurgery, Faculty of Medicine, National University Hospital Damascus University Damascus Syria; ^2^ Syrian Medical Research Group Damascus Syria

**Keywords:** case report, neuroendoscopy, optic disc pallor, pediatric hydrocephalus, suprasellar arachnoid cyst, ventriculocystocisternostomy

## Abstract

Suprasellar arachnoid cysts are uncommon pediatric lesions that may cause obstructive hydrocephalus and optic pathway compromise, often with insidious clinical progression. We report an 11‐year‐old boy who presented with a 7‐year history of progressive gait disturbance and imbalance. Neurological examination was largely normal, and visual acuity was preserved; however, fundoscopic examination revealed bilateral optic disc pallor. Brain magnetic resonance imaging demonstrated a giant cerebrospinal fluid–intensity cyst centered in the third ventricle with inferior extension into the suprasellar and prepontine cisterns, causing functional obstruction of the foramina of Monro and marked ventriculomegaly. The patient underwent endoscopic ventriculocystocisternostomy via a right frontal approach, establishing wide communication between the ventricle, cyst, and basal cisterns. Postoperatively, he remained neurologically intact with early improvement in balance. Transient hypoadrenocorticism was identified and successfully managed with hydrocortisone replacement. This case highlights the potential for delayed presentation of suprasellar arachnoid cysts with preserved visual acuity despite chronic optic pathway injury and supports ventriculocystocisternostomy as a shunt‐sparing treatment option in appropriately selected pediatric patients, particularly when cyst anatomy is favorable and long‐term shunt surveillance cannot be reliably ensured.

## Key Clinical Message

Progressive gait disturbance in children may mask suprasellar arachnoid cyst–related hydrocephalus; optic disc pallor can be present despite preserved visual acuity. When surgical treatment is indicated in appropriately selected patients, endoscopic ventriculocystocisternostomy (VCC) can provide shunt‐sparing CSF diversion and may be particularly advantageous when long‐term follow‐up and shunt surveillance cannot be reliably ensured.

## 1 Introduction

Suprasellar arachnoid cysts (SACs) are uncommon, benign cerebrospinal fluid (CSF) collections arising from developmental abnormalities of arachnoid membranes in the suprasellar/third ventricular region. Although SACs are rare overall, they represent a proportionally larger subgroup of arachnoid cysts in children than in adults, accounting for up to 21% of pediatric arachnoid cysts compared with approximately 9% of adult arachnoid cysts in reported series [1]. Because of their proximity to the optic apparatus, hypothalamic–pituitary axis, and CSF pathways, SACs may present in children with symptoms related to mass effect and obstructive hydrocephalus, including gait disturbance, visual impairment, and endocrine dysfunction [2, 3, 4, 5, 6]. Clinical evolution may be insidious, and neuro‐ophthalmologic findings can reflect chronic injury even when central visual acuity is preserved, underscoring the importance of early recognition and appropriate surveillance [6].

Treatment options include cyst shunting, open microsurgical fenestration, and endoscopic internal diversion. Over the past two decades, neuroendoscopic fenestration has been increasingly adopted because it can achieve decompression with lower surgical morbidity and may avoid long‐term device dependence [2, 3, 4, 5, 7]. Endoscopic strategies include ventriculocystostomy (VC) and ventriculocystocisternostomy (VCC). VCC aims to establish a wide, durable communication between the ventricle, cyst cavity, and basal cisterns—an approach supported by early endoscopic principles favoring dual fenestration and by later pediatric series suggesting greater durability compared with single‐stoma techniques in appropriately selected patients [3, 7, 8].

Nevertheless, management decisions must also account for healthcare delivery realities. Shunt‐based strategies carry risks of infection and malfunction that frequently necessitate ongoing surveillance and revision, which can be challenging in settings with constrained follow‐up capacity [9, 10, 11]. Reports from low‐resource and conflict‐affected environments remain relatively limited despite the practical relevance of definitive, device‐sparing endoscopic solutions in such contexts [11]. We present an 11‐year‐old boy from Syria with a symptomatic SAC and delayed presentation, treated successfully with endoscopic VCC. This case highlights diagnostic considerations, selection of VCC over alternative CSF diversion strategies, and the feasibility of guideline‐concordant neuroendoscopic management where continuity of follow‐up and shunt surveillance cannot be assumed.

## 2 Case History and Examination

An 11‐year‐old boy presented with a 7‐year history of progressive gait disturbance and imbalance, which had gradually worsened over time. There was no history of headache, seizures, visual complaints, endocrine symptoms, head trauma, or previous neurosurgical intervention. His past medical, surgical, and family histories were unremarkable.

On clinical examination, the patient was alert and oriented, with normal higher mental functions. Cranial nerve examination revealed preserved visual acuity of 10/10 bilaterally; however, fundoscopic examination demonstrated bilateral optic disc pallor. Pupillary light reflexes were normal, and extraocular movements were full without diplopia or gaze palsy. Bedside visual field assessment by confrontation did not reveal a gross visual field deficit. Formal automated perimetry and optical coherence tomography were not available in the local resource‐limited setting at the time of evaluation. Motor strength, sensory examination, and cerebellar testing were normal. Gait was broad‐based and unsteady but without focal motor deficit. No dysmorphic features or clinical signs of endocrine dysfunction were noted.

## 3 Investigations

Routine laboratory investigations were within normal limits. Baseline endocrine evaluation, including assessment of pituitary–adrenal and thyroid axes, showed no abnormalities.

Brain magnetic resonance imaging (MRI) demonstrated a large, well‐defined cystic lesion measuring 4.5 × 4.5 × 6.7 cm located within the third ventricle, extending inferiorly into the suprasellar and prepontine cisterns. The lesion was isointense to cerebrospinal fluid (CSF) on all sequences, with a thin, smooth wall and no contrast enhancement, consistent with a suprasellar arachnoid cyst (Figure 1). The cyst caused compression/closure of the foramina of Monro, resulting in marked dilatation of both lateral ventricles with minimal transependymal edema. The inferior third ventricular floor was displaced downward into the suprasellar/prepontine cisternal region. The lesion displaced vessels of the circle of Willis and exerted mass effect on the midbrain and brainstem. The cerebral aqueduct and fourth ventricle were normal. Bilateral tortuosity of the optic nerves with optic nerve sheath dilatation was also observed, consistent with raised intracranial pressure. Together, the clinical and radiological findings indicated a symptomatic SAC driving both obstructive hydrocephalus and cyst‐related mass effect, supporting surgical treatment with a cyst‐directed dual fenestration strategy rather than treatment of hydrocephalus alone.

**FIGURE 1:**
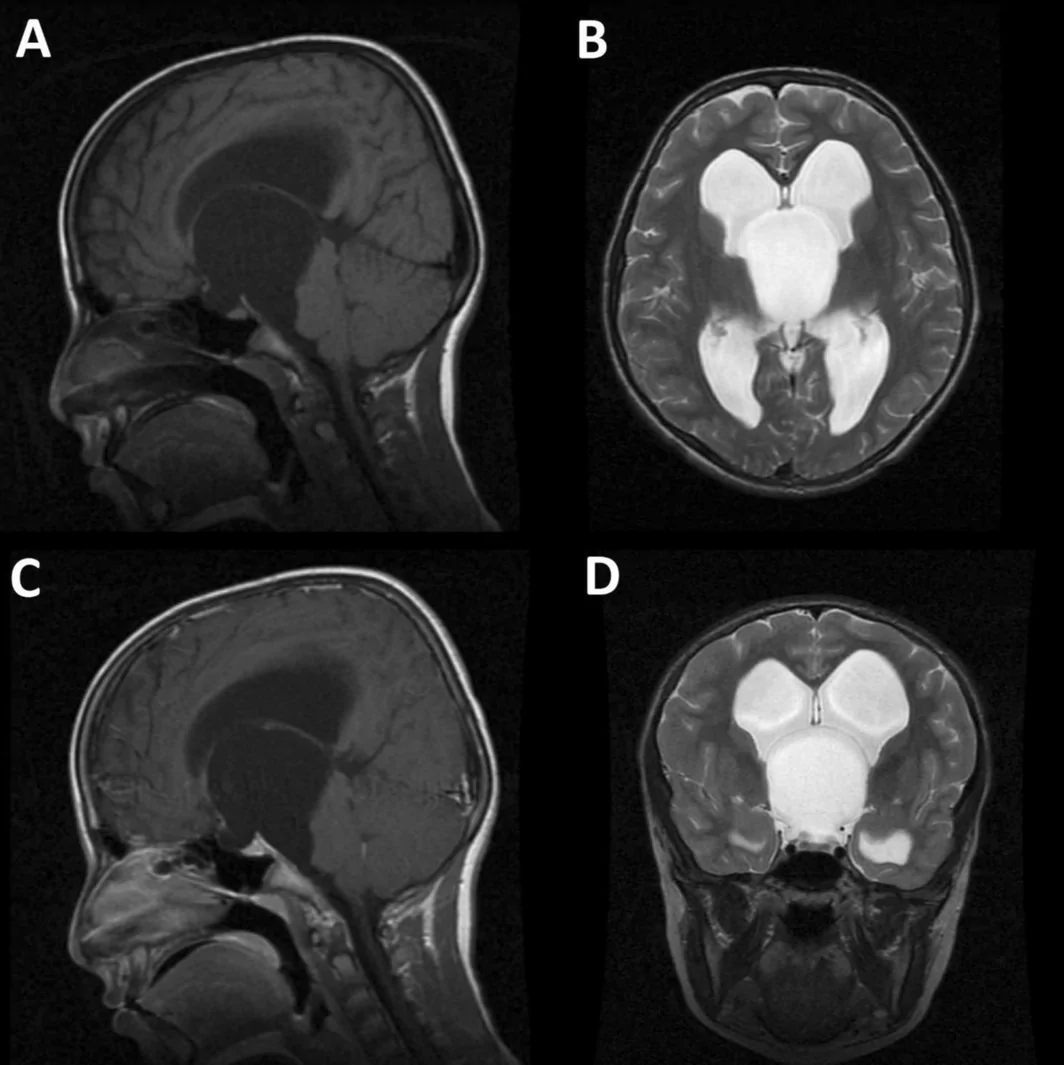
Preoperative brain MRI. (A) Sagittal T1‐weighted image demonstrating a large cystic lesion centered in the third ventricle with inferior extension into the suprasellar region and downward displacement of the third ventricular floor. (B) Axial T2‐weighted image showing marked dilatation of the lateral ventricles consistent with obstructive hydrocephalus caused by a CSF‐intensity cyst. (C) Post‐contrast sagittal T1‐weighted image demonstrating absence of cyst wall enhancement. (D) Coronal T2‐weighted image illustrating suprasellar extension of the cyst with mass effect on adjacent structures.

## 4 Treatment

Under general anesthesia, the patient underwent neuroendoscopic ventriculocystocisternostomy via a right frontal burr hole at Kocher's point. The trajectory was planned to enter the frontal horn of the lateral ventricle and provide a direct route toward the cyst dome. After ventricular entry, the endoscope was advanced under direct visualization using the choroid plexus, septal structures, and the region of the foramen of Monro as anatomical landmarks. The apical cyst membrane was identified and widely fenestrated to establish communication between the lateral ventricle and the cyst cavity (Figure 2A). The endoscope was then advanced into the cyst cavity, and the basal membrane was inspected carefully. Basal fenestration was performed toward the prepontine cistern under continuous direct visualization, with particular attention to avoiding the hypothalamic region, perforating vessels, and basilar artery complex. A wide communication between the ventricular system, cyst cavity, and basal cisterns was achieved (Figure 2B). A temporary external ventricular drain was placed for short‐term postoperative intracranial pressure control and CSF pathway adaptation.

**FIGURE 2:**
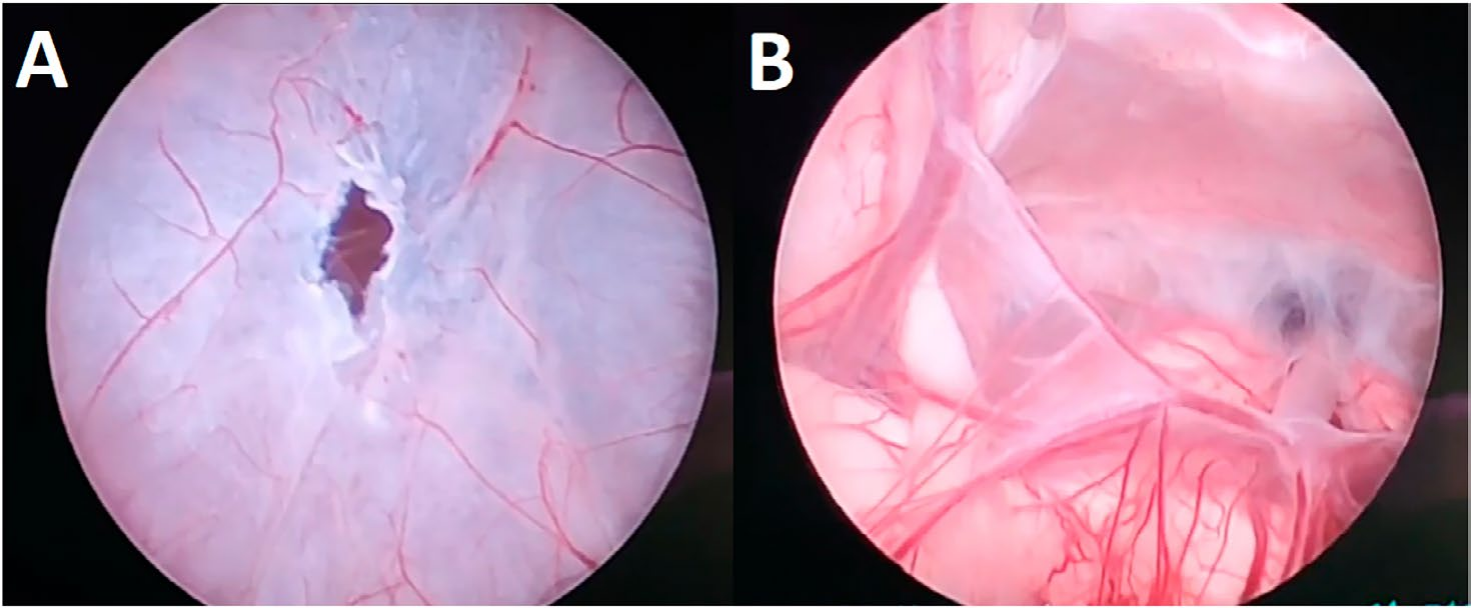
Intraoperative endoscopic views during ventriculocystocisternostomy. (A) Endoscopic view showing fenestration of the apical cyst membrane, establishing communication with the ventricular system. (B) Endoscopic view through the basal cyst opening demonstrating communication with the prepontine cistern and visualization of basal cisternal structures.

## 5 Outcome and Follow‐Up

The patient was monitored in the neurosurgical intensive care unit (ICU) for 24 h, then transferred to the ward. He remained hemodynamically stable with GCS 15/15 and no new neurological deficits developed. Early postoperative CT confirmed appropriate positioning of the fenestration trajectory and absence of hemorrhagic or ischemic complications. Transient hypoadrenocorticism was detected biochemically and managed with hydrocortisone replacement; adrenal function normalized on follow‐up testing. No additional endocrine, visual, or neurological complications were observed. The EVD was removed uneventfully. He was discharged on postoperative Day 4 with normal neurological examination and marked subjective improvement in balance. Outpatient follow‐up was arranged with neurosurgery, ophthalmology, and endocrinology.

At 3‐month follow‐up, the patient remained clinically stable with sustained improvement in balance; postoperative MRI demonstrated reduced cyst size and improved ventricular configuration. Continued clinical, endocrine, and ophthalmologic surveillance, with interval imaging planned at approximately 12 months, was arranged.

## 6 Discussion

Suprasellar arachnoid cysts (SACs) are uncommon lesions that may present with obstructive hydrocephalus, visual pathway compromise, and endocrine disturbance due to their close anatomical relationship with the third ventricular floor, hypothalamus, and optic apparatus [2, 3, 4, 5]. Endoscopic management has become a preferred first‐line strategy in symptomatic SACs because it avoids craniotomy morbidity and, when effective, can obviate lifelong CSF shunt dependence [2, 3, 4, 5]. The main technical debate is whether ventriculocystostomy (VC) alone is sufficient or whether ventriculocystocisternostomy (VCC) provides superior durability by restoring CSF communication across the ventricle–cyst–basal cistern compartments [2, 3]. Although many arachnoid cysts are benign incidental findings, the marked obstructive hydrocephalus, signs of raised intracranial pressure, optic pathway compromise, and progressive gait disturbance in this patient established a clear clinicoradiological indication for treatment rather than observation.

Several features of the present case strengthen its reportable value. The child had a markedly delayed presentation, with 7 years of progressive imbalance and clear radiological signs of chronic obstruction. Bilateral optic disc pallor despite preserved visual acuity and no gross deficit on bedside visual field assessment suggests chronic, partially compensated optic neuropathy. This apparent dissociation may be explained by partial optic atrophy with relative preservation of central papillomacular fibers, allowing Snellen acuity to remain intact despite structural optic nerve injury [12, 13]. In the present case, longstanding hydrocephalus with intracranial hypertension and chronic compression or stretching of the anterior visual pathways may have contributed to anterior visual pathway compromise. Preserved visual acuity alone does not exclude chronic optic nerve injury and should not be used as the sole parameter for neuro‐ophthalmologic assessment or follow‐up, because formal visual field testing may better reflect functional impairment in optic nerve pallor [12]. Therefore, similar patients should undergo formal baseline and longitudinal neuro‐ophthalmologic assessment, ideally including automated perimetry and optical coherence tomography when available, to monitor visual field function and structural optic nerve integrity.

This clinical–radiological mismatch highlights how delayed access to care may allow compensatory functional reserve to persist while structural injury evolves, potentially postponing diagnosis until irreversible damage has occurred. The prolonged diagnostic interval was likely driven primarily by limited access to advanced neuroimaging and specialized pediatric neurosurgical evaluation in the patient's conflict‐affected, resource‐limited local setting. This delay may have been further compounded by the insidious nature of gait imbalance and the absence of headache, seizures, overt visual complaints, or endocrine symptoms. Earlier MRI could potentially have identified obstructive hydrocephalus before optic disc pallor developed and may have allowed earlier intervention.

The choice of endoscopic VCC was justified on anatomical and pragmatic grounds. In this patient, MRI demonstrated foraminal obstruction with marked lateral ventriculomegaly, inferior extension of the cystic third ventricular/suprasellar compartment into the suprasellar and prepontine cisterns, displacement of the circle of Willis vessels, and mass effect on the midbrain and brainstem, while the aqueduct and fourth ventricle remained normal. These findings indicated a combined ventricle–cyst–cistern compartment problem rather than isolated aqueductal obstruction. A dual fenestration strategy was therefore selected to establish communication superiorly between the ventricle and cyst and inferiorly between the cyst and basal cisterns. This approach was intended to decompress both the ventricular and cyst compartments and reduce the likelihood of persistent cyst isolation or isolated stoma reclosure. Early endoscopic work emphasized fenestration into both the ventricle and basal cisterns [8], and pediatric series comparing VC and VCC have suggested lower recurrence or reoperation rates with VCC in appropriately selected patients [2, 3]. Recent single‐center experience continues to support favorable outcomes for neuroendoscopic approaches in SACs [7].

Alternative strategies were considered but were less suitable in this specific anatomical and healthcare‐access context. VC alone is technically simpler and may be appropriate when ventricular decompression and cyst communication are sufficient; however, in large inferiorly extending SACs, VC may not reliably restore communication with the basal cisterns. ETV can be useful when hydrocephalus predominates and cyst‐related mass effect is limited, and combined endoscopic strategies may be appropriate when anatomy permits safe treatment of both obstructive hydrocephalus and cyst compartmentalization. In the present case, however, the large cyst compartment, inferior extension toward the prepontine cistern, distortion of the third ventricular floor, and cyst‐related mass effect favored a cyst‐directed dual fenestration strategy rather than ETV alone. Primary shunting was deliberately avoided because shunts impose device dependence, with clinically meaningful infection and malfunction risks over time [9, 10]. These risks can be amplified when access to urgent revision, sterile resources, and reliable follow‐up is constrained, making a definitive shunt‐sparing endoscopic strategy particularly attractive where continuity of care is uncertain [11]. In such contexts, minimizing dependence on implanted CSF diversion devices represents a pragmatic approach to reducing downstream morbidity rather than merely a technical preference.

Postoperative endocrine considerations are also important. Even when preoperative endocrine testing is normal and early postoperative changes are transient, long‐term follow‐up series show that endocrine abnormalities can evolve over time in SAC patients [6]. This supports structured, longitudinal endocrine follow‐up (in addition to imaging and ophthalmology), rather than concluding success based solely on early postoperative status. Although the early clinical and radiological response was favorable, the 3‐month follow‐up remains short for a pediatric SAC treated endoscopically. Longer‐term surveillance is required to confirm durable cyst decompression and ventricular stability and to monitor for delayed endocrine or neuro‐ophthalmologic sequelae, as well as radiological recurrence.

The treatment strategy described in this case should not be generalized indiscriminately to all pediatric patients with suprasellar arachnoid cysts or across all age groups. Surgical decision‐making should be individualized according to patient age, ventricular size, cyst morphology, membrane configuration, mechanism of hydrocephalus, feasibility of safe endoscopic access, available equipment, and endoscopic expertise. VCC is most applicable when cyst anatomy permits safe fenestration into both the ventricular system and basal cisterns; in other anatomical or clinical settings, VC, ETV, combined endoscopic procedures, or shunt‐based diversion may remain appropriate.

Clinical implications are practical: (i) children with progressive gait disturbance or subtle signs of raised intracranial pressure warrant timely MRI review for treatable obstructive lesions; (ii) optic disc pallor—even with preserved acuity—should trigger expedited treatment and structured neuro‐ophthalmic surveillance; and (iii) in environments where shunt maintenance and follow‐up reliability are limited, VCC is a rational, anatomy‐based option that can reduce long‐term device dependence while restoring CSF pathways in appropriately selected SACs.

## 7 Conclusion

Suprasellar arachnoid cysts may present with prolonged, subtle symptoms despite advanced hydrocephalus. In appropriately selected pediatric patients with favorable cyst anatomy, endoscopic ventriculocystocisternostomy may provide a shunt‐sparing treatment option, particularly when long‐term follow‐up and shunt surveillance are uncertain. Long‐term clinical, radiological, ophthalmologic, and endocrine surveillance remains essential.

## Author Contributions

**Zolfiqar Ibrahem:** conceptualization, data curation, investigation, methodology, supervision, writing – original draft, writing – review and editing. **Abdulrahman Almjersah:** conceptualization, formal analysis, funding acquisition, investigation, methodology, project administration, resources, software, supervision, visualization, writing – original draft, writing – review and editing. **Abdullah Mouselli:** conceptualization, data curation, formal analysis, investigation, methodology, project administration, resources, software, visualization, writing – original draft, writing – review and editing. **Iskandar Ismail Saleh:** conceptualization, data curation, formal analysis, funding acquisition, investigation, methodology, resources, supervision, writing – review and editing. **Basel Alromhain:** conceptualization, investigation, project administration, resources, software, supervision, visualization, writing – review and editing.

## Funding

The authors have nothing to report.

## Ethics Statement

Our institution does not require ethical approval for reporting individual cases. This study was performed in accordance with the Helsinki Declaration of 1964 and its later amendments.

## Consent

Written informed consent for publication of this case report and accompanying images was obtained from the patient's parent/legal guardian in accordance with the journal's patient consent policy. The original signed consent form is retained by the authors and will be provided to the Publisher upon request.

## Conflicts of Interest

The authors declare no conflicts of interest.

## Data Availability

All relevant data supporting the findings of this case report are included within the article. Additional details are available from the corresponding author upon reasonable request.
